# The Gradual Shift of Overweight, Obesity, and Abdominal Obesity Towards the Poor in a Multi-ethnic Developing Country: Findings From the Malaysian National Health and Morbidity Surveys

**DOI:** 10.2188/jea.JE20170001

**Published:** 2018-06-05

**Authors:** Jeevitha Mariapun, Chiu-Wan Ng, Noran N. Hairi

**Affiliations:** Julius Centre University of Malaya, Department of Social and Preventive Medicine, Faculty of Medicine, University of Malaya, Kuala Lumpur, Malaysia

**Keywords:** overweight, obesity, socioeconomic status, developing countries, economic development

## Abstract

**Background:**

Economic development is known to shift the distribution of obesity from the socioeconomically more advantaged to the less advantaged. We assessed the socioeconomic trends in overweight, obesity, and abdominal obesity across a period of significant economic growth.

**Methods:**

We used the Malaysian National Health and Morbidity Survey data sets for the years 1996, 2006, and 2011 to analyze the trends among adults aged 30 years and above. The World Health Organization’s Asian body mass index cut-off points of ≥23.0 kg/m^2^ and ≥27.5 kg/m^2^ were used to define overweight and obesity, respectively. Abdominal obesity was defined as having a waist circumference of ≥90 cm for men and ≥80 cm for women. Household per-capita income was used as a measure of socioeconomic position. As a summary measure of inequality, we computed the concentration index.

**Results:**

Women in Peninsular Malaysia demonstrated patterns that were similar to that of developed countries in which the distributions for overweight, obesity, and abdominal obesity became concentrated among the poor. For women in East Malaysia, distributions became neither concentrated among the rich nor poor, while distributions for men were still concentrated among the rich. Chinese women, particularly from the richest quintile, had the lowest rates and lowest increase in overweight and obesity. All distributions of Chinese women were concentrated among the poor. The distributions of Malay men were still concentrated among the rich, while distributions for Chinese and Indian men and Malay and Indian women were neither concentrated among the rich nor poor.

**Conclusion:**

As the country continues to progress, increasing risks of overweight and obesity among the socioeconomically less advantaged is expected.

## INTRODUCTION

The advancement of Malaysia towards becoming a high-income nation adversely presents a growing challenge of an obesity epidemic. With escalating obesity rates and about half of the adult population currently overweight or obese, Malaysia is now the most overweight and obese nation in the south-east Asian region.^1^^,^^2^ This is alarming because, compared to those with a normal/healthy weight, people who are overweight or obese are at increased risk of major chronic diseases, such as cardiometabolic diseases (heart disease, diabetes, and hypertension) and cancer.^1^^,^^3^^,^^4^ With cardiovascular disease (CVD) being the highest burden of disease and deaths in Malaysia,^4^^,^^5^ efforts to prevent or decrease obesity in the population would be beneficial in reducing cardiovascular-related diseases, premature CVD deaths, other obesity co-morbidities, and accompanying health costs.^1^^,^^3^^,^^6^

The transition of higher obesity prevalence from the rich to poor first emerged in high-income countries and recently in low- and middle-income countries (LMICs). These changes are very much consequences of economic development, which alters the socioeconomic characterization of a country’s obesity distribution.^7^^–^^9^ In low-income countries, obesity is more prevalent among the rich than the poor.^10^^,^^11^ Conversely, in high-income countries, socioeconomic ranking and obesity appears to be inversely associated.^10^^,^^11^ Malaysia is an upper-middle income country progressing to achieve high-income status by the year 2020. The period of rapid economic development in Malaysia began in the late 1980s, particularly in Peninsular Malaysia.^12^ Apart from periods of economic crises in 1997/8 and 2007/8, the gross domestic product (GDP) has been generally rising over the years. We hypothesize that a socioeconomic transition in obesity may very well be occurring in Malaysia too. If this is found to be true, this would mean that, as the country continues to progress, more people from socioeconomically disadvantaged backgrounds would have higher risk of obesity. This would eventually create or raise unfairness and injustice in the local health distribution.^13^

We explored the trends of the socioeconomic inequalities in overweight, obesity, and abdominal obesity among Malaysian adults at multiple time-points across a period of 15 years (1996, 2006, and 2011) using population-based data sets. The GDP per-capita for the years 1996, 2006, and 2011 were USD 4,743.7, USD 6,194.7, and USD 10,427.8, respectively.^14^ The examination of the socioeconomic trends in obesity would enable more accurate projections of prospective obesity trends across the socioeconomic distribution. Most studies on the trends in socioeconomic inequalities in obesity have been conducted in high-income countries. Furthermore, thus far the socioeconomic trends of overweight, obesity, or abdominal obesity have yet to be identified in Malaysia.

Malaysia is geographically comprised of Peninsular Malaysia and East Malaysia, which are separated by the South China Sea. Unlike East Malaysia, over the last 2 decades, economic growth in Peninsular Malaysia has been relatively more intense. Accelerated economic growth has been linked to the widening of health inequalities in a population.^15^^–^^17^ Considering dissimilarities in the pace of development, we examined both geographical regions separately. Past reviews have shown that there are gender differences in the transition of the socioeconomic distribution of obesity, where women demonstrate an earlier shift from a positive to a negative association compared to men as countries evolve economically. Therefore, men and women were also independently assessed. A systematic review by Sobal and Stunkard on the association between socioeconomic position and obesity found that in developed countries, obesity in women was more prevalent among those of lower socioeconomic positions.^18^ This finding was, however, less consistent for men. For developing countries, the reverse was found to be true for both women and men. An update of Sobal and Stunkard’s review by Mclaren almost 2 decades later found smaller differences between the opposing socioeconomic trends of the prevalence of obesity among women of high-income and low-income countries.^19^ This was presumed to be caused by the accelerated pace of globalization over time. Malaysia’s population is primarily made up of three major ethnic groups—namely the Malays (50.1%), Chinese (22.6%), and Indians (6.7%)—besides the indigenous natives, other ethnic minorities, and non-citizens.^20^ Local studies on the prevalence of obesity have shown higher prevalence among Malays and Indians compared to Chinese.^4^^,^^21^ Cultural factors appear to influence dietary and lifestyle behaviors differently.^22^ Furthermore, the Chinese are known to be socioeconomically more advantaged than the other ethnic groups.^23^ For these reasons, as a secondary objective, we examine the socioeconomic distribution of overweight within these three ethnic groups to identify if there are apparent disparities. For this objective, we focus on Peninsular Malaysia, as it is more representative of the national ethnic distribution. A shift of the burden of obesity and its concomitant diseases to the poor would indicate rising health inequities in the country as it moves into a high-income economy.^24^ Essentially, recognizing the demographics of obesity over years of economic development would serve as valuable evidence to policy-makers.

## METHODS

The secondary datasets that were used in our analysis were from three National Health and Morbidity Surveys (NHMS)—1996, 2006, and 2011. These population-based national surveys are conducted “to provide heath related community-based data and information for the Ministry of Health to review health priorities, programme strategies and activities, and planning for allocation of resources”.^25^ The NHMS is a cross-sectional community survey that began in the year 1986. The surveys were conducted in 10-year intervals till year 2006, after which it was held every 4 years focusing on specific health topics and age groups. All the NHMS adopt a two-stage stratified random sampling method. The enumeration block (EB) was the sampling unit for the first stage and the living quarters (LQs) were the second stage units. Response rates were 86.9%, 90.0%, and 88.2% for the years 1996, 2006, and 2011, respectively. For all three years, all respondents in the household were interviewed on questions regarding personal and household information. Questions pertaining to household characteristics were administered to each household. For health outcomes and health- and lifestyle-related behaviors, the age groups that were interviewed differed across the years. For year 1996, only adults aged 30 years and above were interviewed for health outcomes and related behaviors pertaining to cardiovascular risk factors. For years 2006 and 2011, adults aged 18 years and above were interviewed. For year 1996, weight was measured using a daily-calibrated spring balance and height was measured using a measuring tape attached to a steady wall.^26^ Abdominal obesity was not measured in the 1996 NHMS. For years 2006 and 2011, weight was measured using the TANITA Personal Scale HD-319 (Tanita Corporation, Tokyo, Japan), height was measured using the SECA 206 Body Meter (Seca Nihon, Chiba, Japan) and waist circumference was measured using the SECA 200 measuring tape.^25^^,^^27^ All measurements were assessed by trained health personnel.

We analyzed the socioeconomic trends in overweight and obesity of adults aged 30 years and older, with complete data for household income and body mass index (BMI) for the years 1996, 2006, and 2011 and abdominal obesity for the years 2006 and 2011. The World Health Organization-recommended BMI cut-off point for public health action of ≥23.0 kg/m^2^ was used to define overweight and ≥27.5 kg/m^2^ for obesity.^28^ Abdominal obesity was defined as having a waist circumference of ≥90 cm for men and ≥80 cm for women. As a measure of socioeconomic position, household per-capita income (PCI), which was self-reported household income accounting for household size, was used.

Age-adjusted prevalence of overweight was calculated using the direct standardization method. Age-gender standardizing proportions of 5-year age groups (0–4, …, 75–79, and ≥80 years) of the population of Malaysia were applied to the populations of Peninsular Malaysia and East Malaysia. Socioeconomic inequality in overweight/obesity and obesity in the population were measured using the concentration index (CI).^29^ The strengths of the CI as a measure of socioeconomic inequality in health is that, unlike some relative measures that demonstrate differences based on the extremes of socioeconomic position, the CI reflects the experiences of the entire distribution.^30^ Besides including the socioeconomic aspect to inequalities in health, the CI is also sensitive to variations in the distribution across the groups.^30^ The measure of socioeconomic position is denoted by the respective individual’s household PCI. The degrees of socioeconomic inequality^31^ in overweight and obesity are quantified by the concentration index (CI), which ranges from the values of −1 to 1.^32^ The CI is defined as:

$$CI=\frac{2}{\mu }\text{cov}({\text{h}}_{\text{i}},{\text{r}}_{\text{i}})$$

where h_i_ is the health variable of individual i, r is the fractional ranking of socioeconomic position, μ is the mean of h and the covariance between h_i_ and r_i_ is denoted by cov(h_i_, r_i_).^32^ An index of zero indicates no socioeconomic related inequality in overweight and obesity, a negative index indicates that overweight and obesity are concentrated among poorer individuals, and a positive index indicates that overweight and obesity are concentrated among richer individuals.^32^ Considering that the health outcomes were binary variables, to enable comparison over time, the CI was normalized by dividing CI by 1 − μ.^33^ All analyses were conducted using STATA version 12.1 (StataCorp, College Station, TX, USA).

### Ethical approval

All individual level data acquired for this study were anonymized data obtained from the Institute of Public Health, Ministry of Health Malaysia. Ethical approval for this study was obtained from the Medical Research Ethics Committee (MREC), Ministry of Health Malaysia (NMRR-14-1350-19314).

## RESULTS

**Figure 1.**
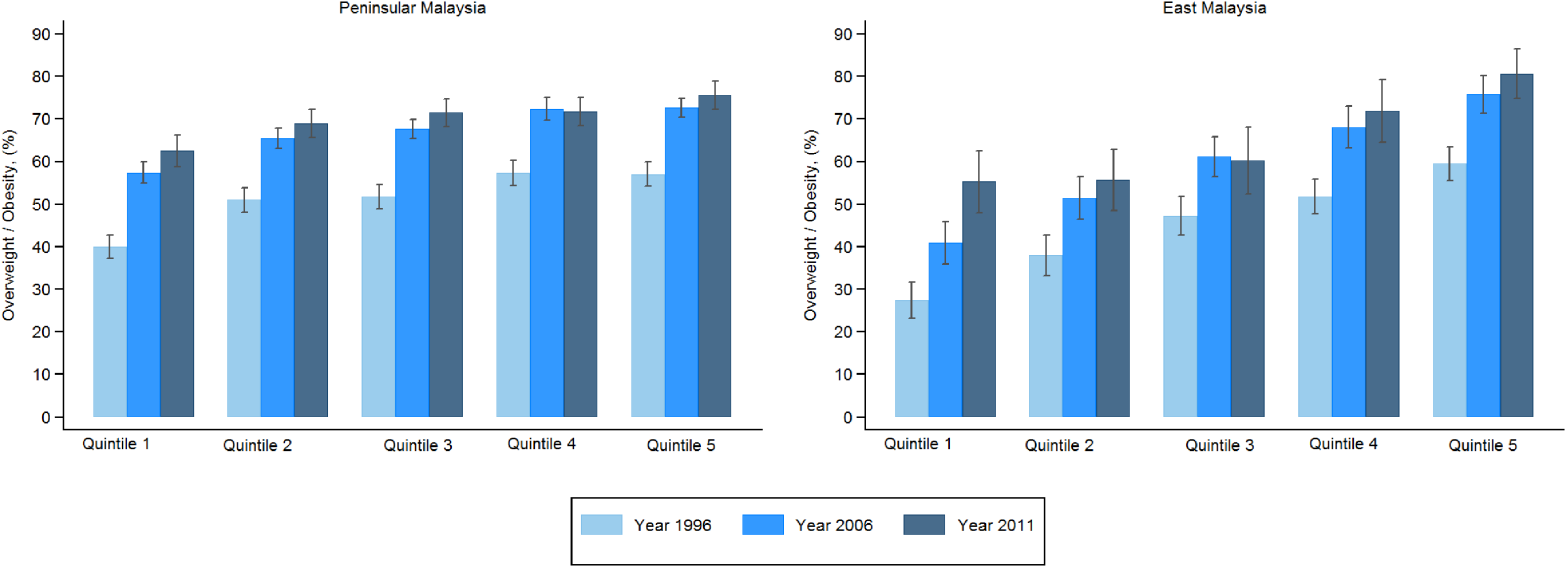
Age-adjusted prevalence of overweight of men by socioeconomic quintiles and region. All quintiles have a significant increasing trend by year (*P* < 0.001)

**Figure 2.**
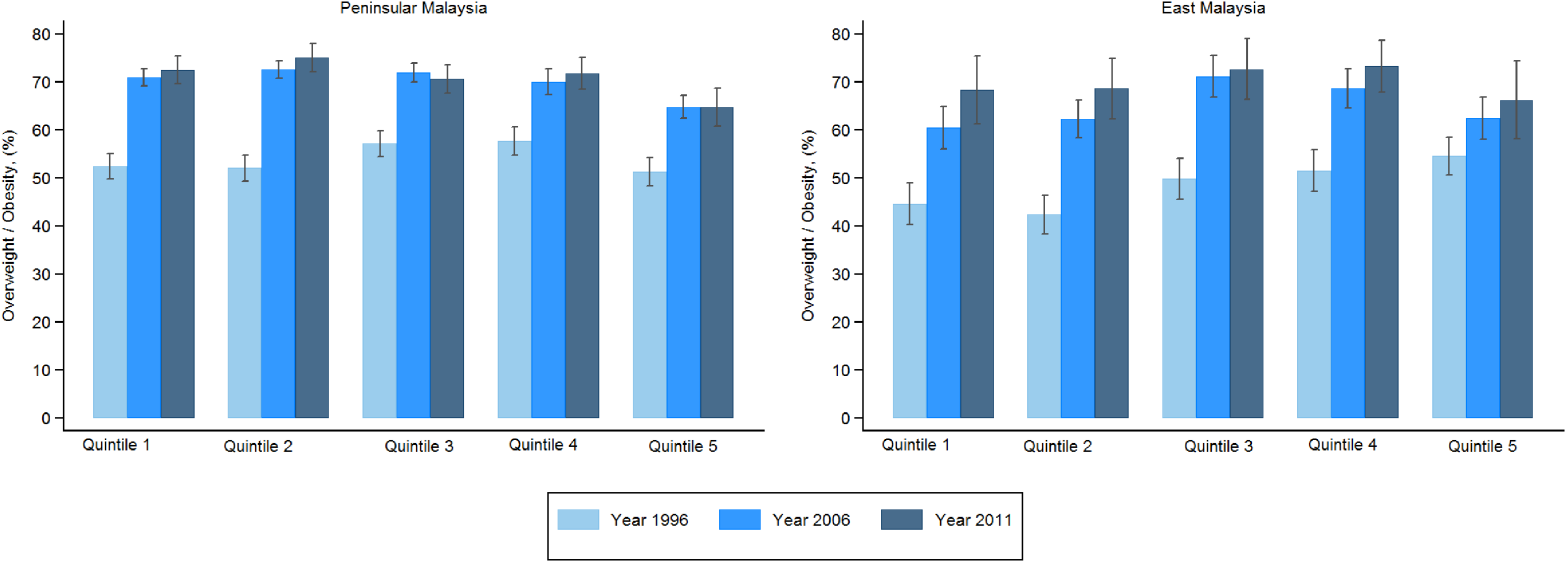
Age-adjusted prevalence of overweight of women by socioeconomic quintiles and region. All quintiles have a significant increasing trend by year (*P* < 0.001)

Figure 1 and Figure 2 show age-adjusted prevalence of overweight by socioeconomic quintiles and years for Peninsular Malaysia and East Malaysia for men and women, respectively. For men, all three surveys mostly showed increasing patterns for overweight across the socioeconomic quintiles, with the poorest quintile having the lowest rates. These results were similar for both the Peninsular and East Malaysia. Between the two regions, the rates for the poorer quintiles were lower for all the years for East Malaysia than for Peninsular Malaysia. For women in East Malaysia, the pattern and trends were almost similar to that of men. The only difference was for the richest quintile, where there was a change from having the highest rates in overweight in 1996 to the lowest rates in 2011. For women in Peninsular Malaysia, the patterns and trends were less consistent. For all three surveys, the richest quintile had the lowest rates of overweight compared to all other quintiles. For the other four quintiles, overweight rates increased across the quintiles for 1996 but levelled out for 2006 and 2011.

**Figure 3.**
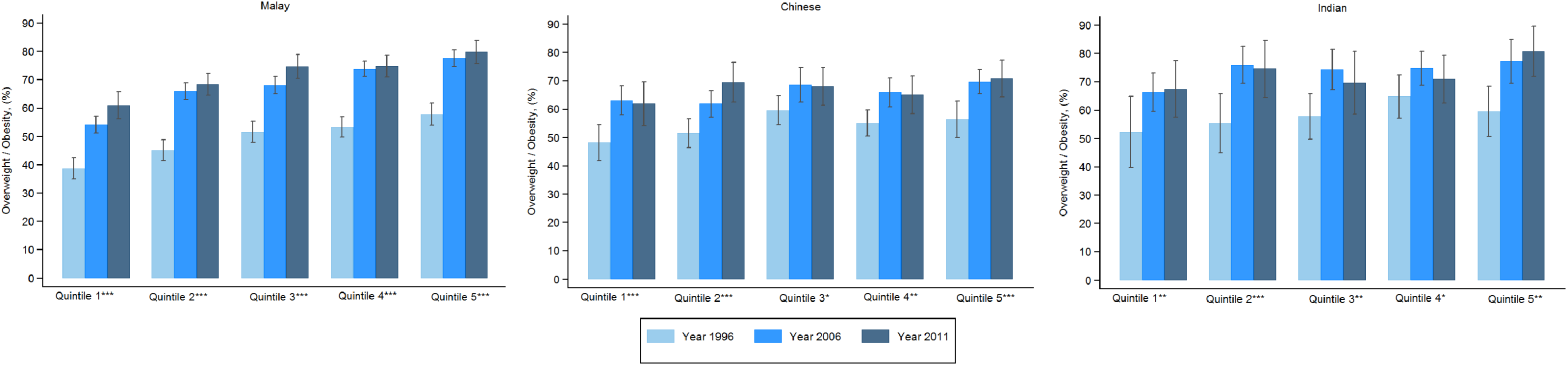
Age-adjusted prevalence of overweight of men by socioeconomic quintiles and ethnicity for Peninsular Malaysia. Quintiles have a significant increasing trend by year if ^*^ (*P* < 0.05), ^**^ (*P* < 0.01), ^***^ (*P* < 0.001).

**Figure 4.**
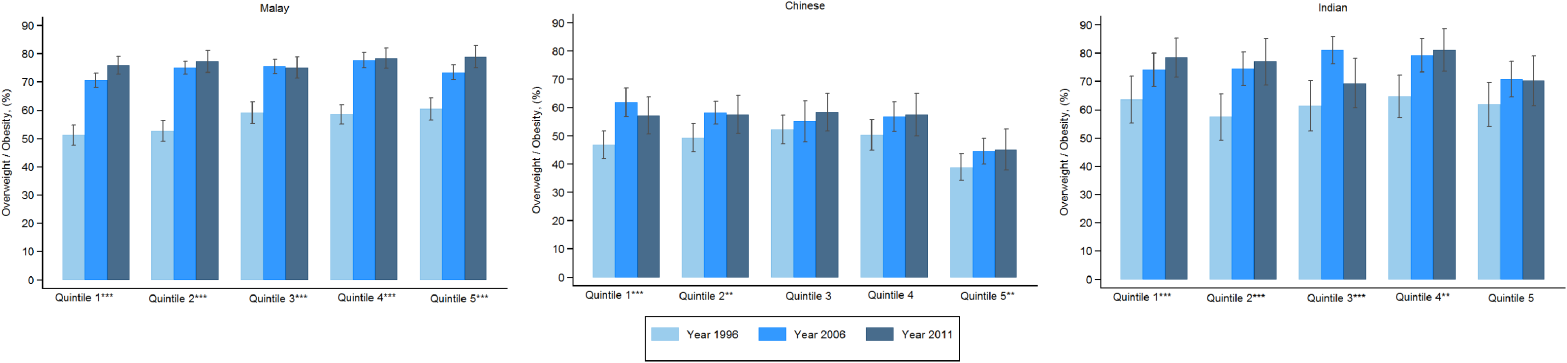
Age-adjusted prevalence of overweight of women by socioeconomic quintiles and ethnicity for Peninsular Malaysia. Quintiles have a significant increasing trend by year if ^*^ (*P* < 0.05), ^**^ (*P* < 0.01), ^***^ (*P* < 0.001).

Figure 3 and Figure 4 show age-adjusted prevalence of overweight by quintiles and years for Malays, Chinese, and Indians in Peninsular Malaysia for men and women, respectively. The Chinese had relatively lower prevalence of obesity than the Malays and Indians. For men, for most cases there were increasing trends in overweight for all three ethnic groups. Malay men experienced the highest increase in overweight. For women, the prevalence was the lowest for the Chinese, especially Chinese women from the richest quintile. The increase in overweight across the years was also the lowest for Chinese women.

Table 1 provides a description of the income distribution of the population by gender, region, and ethnicity. For all years, the median household PCI was higher in Peninsular Malaysia compared to East Malaysia. Chinese had the highest median household PCI, followed by the Indians and then Malays. Table 2 shows the normalized concentration indices and the 95% confidence intervals for overweight, obesity, and abdominal obesity by region, gender, and year of study. The findings show that at all three time-points, the distributions of overweight, obesity, and abdominal obesity for men in both geographical regions were concentrated among the rich. Distributions of obesity and abdominal obesity among men in Peninsular Malaysia and abdominal obesity among men in East Malaysia showed trends of becoming less concentrated among the rich. The normalized concentration index of obesity for men in Peninsular Malaysia decreased from 0.1029 (95% confidence interval, 0.0639–0.1419) in 1996 to 0.0994 (95% confidence interval, 0.0705–0.1284) in 2006 and to 0.0841 (95% confidence interval, 0.0475–0.1206) in 2011. For East Malaysia, the distributions of overweight, obesity, and abdominal obesity among men were relatively more concentrated among the rich than Peninsular Malaysia. For women in Peninsular Malaysia, the negative values of the normalized concentration indices and their respective confidence intervals indicate that the distributions for overweight, obesity, and abdominal obesity had already been concentrated among the poor in year 2006. The distribution for overweight showed a significant trend of becoming more concentrated among the poor. The normalized concentration index of overweight for women in Peninsular Malaysia changed from a distribution that was neither concentrated among the rich nor poor in 1996 (0.0072; 95% confidence interval, −0.0182 to 0.0325) to a distribution concentrated among the poor in 2006 (−0.0418; 95% confidence interval, −0.0661 to −0.0174) and 2011 (−0.0457; 95% confidence interval, −0.0786 to −0.0128). For East Malaysian women, the distributions of overweight, obesity, and abdominal obesity appear to have shifted from distributions concentrated among the rich to distributions that were neither concentrated among the rich nor poor. The distribution for overweight showed a significant trend of becoming less concentrated among the rich. The distribution of overweight changed from a distribution concentrated among the rich in 1996 (0.2865; 95% confidence interval, 0.2431–0.3299) to a distribution that was neither concentrated among the rich nor poor in 2011 (0.0152; 95% confidence interval, −0.0595 to 0.0899).

**Table 1.** Monthly household per-capita income (MYR) by region and ethnicity

Gender	Region	Ethnicity	Year 1996		Year 2006		Year 2011	
		
			*n*	Income	*n*	Income	*n*	Income
Male	Peninsular	Overall	7,051	257 (139–500)	8,160	375 (200–750)	4,611	675 (350–1,200)
		Malay	4,045	200 (113–364)	4,998	300 (163–575)	2,967	594 (320–1,071)
		Chinese	1,963	440 (240–784)	1,928	600 (333–1,000)	1,016	927 (500–1,600)
		Indian	653	313 (171–553)	759	450 (257–800)	420	743 (411–1,223)
	East	Overall	2,819	177 (75–360)	2,209	200 (100–470)	968	441 (234–875)
Female	Peninsular	Overall	7,651	220 (112–433)	9,794	360 (188–667)	5,331	575 (296–1,056)
		Malay	4,393	175 (91–328)	6,032	300 (150–533)	3,342	500 (260–957)
		Chinese	2,155	350 (183–640)	2,168	500 (300–1,000)	1,173	820 (400–1,400)
		Indian	742	264 (143–450)	1,081	400 (238–712)	564	575 (300–1,047)
	East	Overall	2,819	177 (75–360)	2,530	200 (100–455)	1,079	400 (175–800)

MYR, Malaysian Ringgit; *n*, number; values for income are median (interquartile range); overall includes other minority ethnic groups and non-citizens apart from Malay, Chinese & Indian.

**Table 2.** Normalized concentration indices of adult overweight, obesity, and abdominal obesity by region

Region	Gender	Risk factor	Year 1996		Year 2006		Year 2011		*P* value
		
			CI	(95% confidence interval)	CI	(95% confidence interval)	CI	(95% confidence interval)	
Peninsular	Male	Overweight	0.1337	(0.1073, 0.1600)	0.1662	(0.1398, 0.1927)	0.1490	(0.1124, 0.1857)	0.062
		Obesity	0.1029	(0.0639, 0.1419)	0.0994	(0.0705, 0.1284)	0.0841	(0.0475, 0.1206)	0.434
		Abdominal obesity	—	—	0.1225	(0.0968, 0.1481)	0.0740	(0.0402, 0.1078)	0.003^*^
	Female	Overweight	0.0072	(−0.0182, 0.0325)	−0.0418	(−0.0661, −0.0174)	−0.0457	(−0.0786, −0.0128)	0.025^*^
		Obesity	−0.0407	(−0.0723, −0.0091)	−0.0530	(−0.0764, −0.0297)	−0.0452	(−0.0763, −0.0142)	0.941
		Abdominal obesity	—	—	−0.0555	(−0.0784, −0.0326)	−0.0535	(−0.0861, −0.0210)	0.450
East	Male	Overweight	0.2669	(0.2265, 0.3074)	0.3045	(0.2564, 0.3526)	0.2281	(0.1533, 0.3029)	0.003^*^
		Obesity	0.2137	(0.1487, 0.2788)	0.2523	(0.1936, 0.3111)	0.2244	(0.1390, 0.3097)	0.825
		Abdominal obesity	—	—	0.2975	(0.2459, 0.3491)	0.2076	(0.1302, 0.2851)	0.023^*^
	Female	Overweight	0.2865	(0.2431, 0.3299)	0.0523	(0.0060, 0.0987)	0.0152	(−0.0595, 0.0899)	<0.001^*^
		Obesity	0.2271	(0.1580, 0.2963)	0.0289	(−0.0201, 0.0780)	0.0403	(−0.0360, 0.1166)	<0.001^*^
		Abdominal obesity	—	—	0.0554	(0.0095, 0.1013)	0.0269	(−0.0462, 0.1000)	0.441

CI, Concentration Index; ^*^statistically significant differences across the years.

Table 3 shows the normalized concentration indices by ethnicity and gender for years 1996, 2006, and 2011 for Peninsular Malaysia. For Chinese and Indian men, the distribution for overweight had shifted from being concentrated among the rich in 1996 and 2006 to being neither concentrated among the rich nor poor in 2011. The distributions for Malay men, however, were still concentrated among the rich. Overweight and obesity distributions for men from all three ethnic groups showed inconsistent trends across the years. For abdominal obesity, the distribution became less concentrated among the rich from year 2006 to 2011. For Chinese women, the distributions for overweight, obesity, and abdominal obesity had been already concentrated among the poor in 1996, although trends across the years were inconsistent. The distributions of overweight, obesity and abdominal obesity for Chinese women had the lowest values for the normalized concentration indices, indicating that these distributions have the highest concentration among the poor relative to the other categories. The normalized concentration indices were −0.0646 in 1996, −0.1092 in 2006 and −0.0785 in 2011 for overweight, −0.0857 in 1996, −0.1423 in 2006 and −0.1392 in 2011 for obesity and −0.1423 in 2006 and −0.1149 in 2011 for abdominal obesity. For Malay women, the distributions for overweight and obesity were concentrated among the rich while the distribution of abdominal obesity was neither concentrated among the rich nor poor. The distribution for overweight showed a significant trend of becoming less concentrated among the rich. For Indian women, all distributions were neither concentrated among the rich nor poor, indicating no evidence of inequality. The distribution of obesity for Malay and Indian women showed an unexpected trend of becoming more concentrated among the rich; however, this trend was not significant.

**Table 3.** Normalised concentration indices of adult overweight, obesity, and abdominal obesity by ethnicity, Peninsular Malaysia

Gender	Risk Factor	Ethnicity	Year 1996		Year 2006		Year 2011		*P* value
		
			CI	(95% confidence interval)	CI	(95% confidence interval)	CI	(95% confidence interval)	
Men	Overweight	Malay	0.1586	(0.1242, 0.1931)	0.2300	(0.1962, 0.2637)	0.2195	(0.1732, 0.2658)	0.258
		Chinese	0.0817	(0.0314, 0.1320)	0.0912	(0.0376, 0.1448)	0.0695	(−0.0079, 0.1470)	0.708
		Indian	0.0996	(0.0092, 0.1901)	0.1038	(0.0135, 0.1942)	0.0505	(−0.0671, 0.1680)	0.563
	Obesity	Malay	0.1493	(0.0978, 0.2008)	0.1763	(0.1406, 0.2120)	0.1410	(0.0961, 0.1859)	0.435
		Chinese	0.0270	(−0.0474, 0.1013)	0.0772	(0.0127, 0.1418)	−0.0120	(−0.0966, 0.0726)	0.327
		Indian	0.0585	(−0.0635, 0.1804)	0.0218	(−0.0708, 0.1143)	0.0795	(−0.0332, 0.1922)	0.798
	Abdominal Obesity	Malay	—	—	0.1807	(0.1480, 0.2134)	0.1445	(0.1028, 0.1862)	0.020^*^
		Chinese	—	—	0.0151	(−0.0374, 0.0676)	−0.0642	(−0.1364, 0.0079)	0.105
		Indian	—	—	0.0258	(−0.0564, 0.108)	−0.0248	(−0.1406, 0.0909)	0.497
Women	Overweight	Malay	0.0747	(0.0413, 0.1081)	0.0615	(0.0291, 0.0939)	0.0542	(0.0083, 0.1002)	0.035^*^
		Chinese	−0.0646	(−0.1117, −0.0175)	−0.1092	(−0.1561, −0.0623)	−0.0785	(−0.1428, −0.0143)	0.624
		Indian	0.0403	(−0.0439, 0.1245)	0.0189	(−0.0598, 0.0977)	−0.0666	(−0.1779, 0.0447)	0.300
	Obesity	Malay	0.0412	(0.0014, 0.0809)	0.0421	(0.0131, 0.0712)	0.0432	(0.0040, 0.0823)	0.885
		Chinese	−0.0857	(−0.1555, −0.0158)	−0.1423	(−0.2002, −0.0843)	−0.1392	(−0.2190, −0.0595)	0.533
		Indian	−0.0513	(−0.1471, 0.0444)	−0.0225	(−0.0916, 0.0466)	−0.0170	(−0.1133, 0.0794)	0.723
	Abdominal Obesity	Malay	—	—	0.0209	(−0.0085, 0.0503)	0.0415	(−0.0013, 0.0844)	0.685
		Chinese	—	—	−0.1423	(−0.1881, −0.0965)	−0.1149	(−0.1786, −0.0512)	0.230
		Indian	—	—	−0.0416	(−0.1177, 0.0344)	−0.1035	(−0.2325, 0.0255)	0.677

CI, Concentration Index; ^*^statistically significant differences across the years.

## DISCUSSION

To the best of our knowledge, this is the first study in Malaysia that assessed the trends of socioeconomic disparities in adult overweight and obesity. Women in Peninsular Malaysia demonstrated patterns that were similar to that of developed countries in which the richest quintile had the lowest rates of overweight and distributions for overweight, obesity, and abdominal obesity were already concentrated among the poor in 2006. For women of East Malaysia, although the distributions of overweight, obesity, and abdominal obesity were not yet concentrated among the poor, they showed a shift from being concentrated among the rich to being neither concentrated among the rich nor poor.

These findings complement the existing literature on the trends of socioeconomic inequalities in obesity, which has generally shown a transition from rich to poor as the economy evolves.^7^^–^^9^^,^^19^ A recent systematic review looking at obesity and socioeconomic position reported consistent trends of overweight and obesity moving towards the poor in developing countries.^8^ These findings correlate with that of earlier reviews on growing economies.^7^^,^^18^^,^^19^

Distributions for men demonstrated patterns similar to that in LMICs. Distributions of overweight, obesity, and abdominal obesity for men were still concentrated among the rich, with richer socioeconomic quintiles having higher rates than poorer quintiles. However, distributions of abdominal obesity for men from both regions and obesity for men in the Peninsular became less concentrated among the rich across the years. In developing countries of middle-income and low-income economies, overweight and obesity appears to be more prevalent among men of higher socioeconomic ranking.^7^ Men from lower socioeconomic backgrounds have relatively more physically demanding occupations, such as manual jobs, unlike the more sedentary white-collar jobs of more socially advantaged men.

The distributions of overweight, obesity, and abdominal obesity were relatively more concentrated among the rich in East Malaysia than in Peninsular Malaysia. These differences are reflections of the dissimilarities in the pace of both regions’ development trajectories. In comparison to Peninsular Malaysia’s rapid economic growth in the past few decades, development in East Malaysia has been more gradual. Popkin et al reported that faster growth of the GDP appears to accelerate the increase of overweight and obesity among the socially less advantaged.^34^ This corresponds with the findings of higher prevalence of obesity among the rich in low-income countries and among the poor in high-income countries.^34^

Among the major ethnic groups of the Peninsular, the Chinese were socioeconomically more advantaged, followed by the Indians and then Malays. Chinese women, particularly from the richest quintile, had the lowest rates and lowest increase in overweight and obesity. The distributions for overweight, obesity, and abdominal obesity were all concentrated among the poor across the years. The distributions for overweight, obesity, and abdominal obesity of Malay men were still concentrated among the rich, while most of the distributions for Chinese and Indian men changed from being concentrated among the rich to being neither concentrated among the rich nor poor. Distributions for Indian women remained neither concentrated among the rich nor poor across the years, indicating no evidence of inequality. The progressive transition of the distributions of overweight and obesity from the rich to poor, which was more apparent among Chinese women in Peninsular Malaysia, has also been portrayed among women in wealthier developing countries.^35^ Chinese women are socioeconomically more advantaged compared to Malay and Indian women. Therefore, it is reasonable to assume that the distributions for Chinese women are comparable to that of women in high-income countries. Women of more developed countries are known to be more concerned about their body image and weight-gain.^36^^,^^37^ As the country continues to progress, we expect both men and women from all ethnic groups to eventually portray similar patterns to what is observed of Chinese women of Peninsular Malaysia. More lower-income individuals will succumb to obesity, and more higher-income individuals will prevent it. In Singapore, which has ethnic groups similar to Malaysia, the prevalence of obesity is shown to be inversely associated to the socioeconomic ranking of ethnic groups. Obesity rates are the highest among the Malays, who earn the least; followed by the Indians, who earn more than the Malays; and lowest among the Chinese, who earn the most.^38^^,^^39^

As seen from our findings, as well as that of other middle and high-income countries, increasing obesity rates in Malaysia is postulated to be eventually more of a problem for the socioeconomically less advantaged. One major reason is the availability and affordability of healthier foods, which influences the lower value food choices preferred by the lower-income group, as opposed to the healthier food varieties, which are more expensive and less energy-dense.^40^^,^^41^ Furthermore, the decrease in the levels of daily physical activity are more significant among the lower-income group, who previously were associated with occupations requiring higher energy expenditures.^8^ On the contrary, the higher-income groups, who are socially more advantaged, generally have higher levels of education, which enables them to have better knowledge and understanding of the importance of healthier diets and active lifestyles. This is especially applicable for women of high socioeconomic status, where the rising trend of obesity appears to be reversed. For instance, there has been a significant decline in obesity among urban women of high income status in Brazil and Scandinavian populations.^42^^,^^43^ This is thought to be brought about by the practice of healthy eating habits and active lifestyles.

Heart disease is currently the leading cause of premature deaths in the country.^5^ It has been reported that a socioeconomic gradient in premature mortality exists in Malaysia, where socioeconomically less advantaged districts were found to have higher rates of premature mortality.^44^ As explained in the introduction, overweight and obesity are associated with higher risks of cardiovascular-related diseases.^45^ Therefore, as hypothesized, if the distribution in obesity becomes more concentrated among the poor, the gradient in premature mortality would widen, resulting in higher health inequity in the country. The influence of socioeconomic position on health can be more clearly understood by the effect it has on material, behavioral, and psychosocial factors, which are the intermediary determinants in the pathway of socioeconomic position and health.^46^ Lower socioeconomic groups are less advantaged in the sense that they have poorer living conditions/environments and have less control over their lives, which makes them more susceptible to stress, encourages unfavorable health behaviors, and negatively affects health.^47^

This study has its strengths and limitations. Our analysis used large samples drawn from nationally representative population-based data sets over a period of 15 years. This rich source of data enables us to show a comprehensive picture of the prevalence and patterns of socioeconomic inequalities and temporal trends in overweight and obesity among Malaysian adults. Use of large national datasets enables us to capture the ethnic minorities, as well as people who are poor and socially isolated. Evidence on these people is scarce because of the difficulty in capturing data through regular sampling surveys. Data for abdominal obesity was not measured for the year 1996, leading to only a depiction of the distribution at two points in time. We did not use weighting for non-response, which may mean under-representation of socioeconomic groups who are less likely to respond.

Basically, the trends show that, as we continue to progress economically, the poor are becoming increasingly burdened by the rising rates of obesity and, consequently, many other related diseases and health conditions. The last year of our analysis was for the year 2011. Assuming similar trends are at the present, the distribution of overweight and abdominal obesity is expected to be skewed further towards the poor. This would eventually have adverse ramifications on the socioeconomic distribution of health, where there would be growing unfairness and injustice in the distribution of health and life expectancy in the country.^48^ As Malaysia works towards achieving a high-income status, it is essential to consider the well-being of the socioeconomically less advantaged population.

Our findings aimed to establish evidence of the existence of health inequality, as this is the first step in addressing health inequality. Further evidence is needed in terms of the differential responses to interventions or practice focusing on inequalities in overweight to reduce the gap between the rich and poor. Future research on other socioeconomic factors influencing obesity, such as education level and occupation, could provide greater understanding of the socioeconomic trends in overweight and obesity in Malaysia. It is important to ensure that overweight interventions acknowledge the potential importance of social patterning and assure that proposed interventions do not further widen these inequalities. We propose that health inequality assessment become a routine with which any practice/policy reforms can be evaluated from the perspective of health inequalities. Addressing inequities in the distribution of obesity should involve actions and services that are universal and with a scale and intensity proportionate to the degree of need.^49^ This would prevent an increased burden on the health system and incurring health costs. Actions taken to obtain a more equitable health distribution would also improve overall population health, which would consequently facilitate a stronger economy.
